# Socioeconomic inequalities in obesity among older adults in the Astana region of Kazakhstan: evidence of a reversed socioeconomic gradient

**DOI:** 10.3389/fpubh.2026.1895120

**Published:** 2026-07-10

**Authors:** Anara Zhumadilova, Tolkyn Zhakupova, Alibek Kossumov, Almagul Kushugulova, Zhaxybay Zhumadilov, Graciela Muniz-Terrera, Zelalem Teka Haile, Adil Supiyev

**Affiliations:** 1 Ohio University Heritage College of Osteopathic Medicine, Ohio University, Athens, OH, United States; 2 Astana Medical University, Astana, Kazakhstan; 3 National Laboratories Astana, Nazarbayev University, Astana, Kazakhstan; 4 Nazarbayev University School of Medicine, Astana, Kazakhstan; 5 Ohio University Heritage College of Osteopathic Medicine, Ohio University, Dublin, OH, United States

**Keywords:** Central Asia, Kazakhstan, obesity, overweight, socioeconomic factors

## Abstract

**Introduction:**

There is limited information on cardiometabolic risk factors in Central Asia. This study examined the prevalence of overweight, obesity, and central obesity and assessed socioeconomic and demographic correlates among older adults in urban and rural areas of the Astana region of Kazakhstan.

**Methods:**

This cross-sectional study included 977 adults aged 50 to 74 years recruited from urban (Astana) and rural (Akmol) sites in the Astana region of Kazakhstan. Multivariable binary logistic regression analyses with three sequential models were conducted to examine associations between obesity measures, socioeconomic characteristics, and cardiometabolic conditions. Robust standard errors were used throughout.

**Results:**

Overall, 37% of participants were overweight, 44% were obese, and 72% had central obesity. Overweight and central obesity were more prevalent in men, while BMI-defined obesity was more common in women (52% vs. 34%). All three obesity measures were significantly and consistently associated with diabetes, hypertension, and hypercholesterolemia across all models. Rural residence was strongly protective against central obesity across all models (fully adjusted OR, 0.43; 95% CI, 0.30–0.64). Greater household asset ownership was positively associated with overweight and obesity in a dose–response fashion, with a significant linear trend confirmed for both outcomes (p for trend = 0.038 and *p* = 0.002, respectively), suggesting a reversed socioeconomic gradient consistent with an early-stage nutrition transition. Educational attainment showed a more complex pattern, with lower education associated with higher odds of obesity in the fully adjusted model. Older age was associated with greater odds of central obesity.

**Conclusion:**

Obesity is highly prevalent among older adults in the Astana region of Kazakhstan. In contrast to the inverse socioeconomic gradient commonly observed in high-income settings, household asset ownership showed a reversed gradient: more affluent participants had significantly higher odds of obesity, whereas lower educational attainment was associated with higher obesity risk. The strong associations between all obesity measures and cardiometabolic conditions underscore the urgent need for integrated obesity and cardiometabolic disease prevention strategies. These findings highlight the need for context-specific public health approaches that account for the distinct socioeconomic patterning of obesity in Kazakhstan.

## Introduction

Obesity has emerged as one of the important public health challenges, with prevalence increasing substantially across both high-income and low and middle-income countries over recent decades (1, 2). Once considered a concern of affluent nations, overweight and obesity are now increasingly prevalent in developing and transitional economies due to rapid urbanization, nutritional transitions, reduced physical activity, and changing dietary patterns (3, 4). Excess adiposity is a major risk factor for several cardiometabolic conditions, including hypertension, diabetes, dyslipidemia, and cardiovascular disease, all of which contribute substantially to premature mortality and disability.

Central Asia has experienced a growing burden of obesity and related noncommunicable diseases in recent years, with prevalence in some countries approaching that observed in high-income settings (5, 6). Among countries in the region, Kazakhstan has one of the highest reported obesity prevalence rates at 23.7%, followed closely by Kyrgyzstan, Uzbekistan, and Turkmenistan, each with comparable prevalence rates of 15.5, 15.2, and 13.2%, respectively. Moreover, a significant gender disparity in obesity prevalence is persistent, with Kazakhstan having the widest gap at nearly 9%, followed by Kyrgyzstan and Uzbekistan, while Turkmenistan records the smallest gap at 1.6%. Given the strong relationship between obesity and cardiometabolic disorders, the rising prevalence of obesity may contribute significantly to the high cardiovascular disease burden observed in the region (7, 8). These trends are likely influenced by ongoing economic, social, and nutritional transitions that have altered lifestyle behaviors, dietary intake, and physical activity patterns across urban and rural populations (9, 10).

The socioeconomic distribution of obesity in transitional settings may differ from patterns commonly observed in high-income countries. In many high-income settings, obesity disproportionately affects socioeconomically disadvantaged populations (11, 12). However, emerging evidence from low- and middle-income and transitional economies suggests that obesity may instead be more prevalent among individuals with higher socioeconomic status, reflecting a reverse socioeconomic gradient (13, 14). Despite growing recognition of obesity as a public health concern in Central Asia, there remains limited individual-level evidence examining the socioeconomic patterning of obesity in Kazakhstan, particularly among older adults and across urban and rural populations.

Understanding obesity among older adults is especially important because excess adiposity in later life is strongly associated with increased risk of chronic diseases, reduced quality of life, and mortality (15–17). Importantly, the study population represents the last generation to have lived their entire formative years under Soviet-era nutritional and socioeconomic conditions, born between approximately 1938 and 1965. Following the dissolution of the USSR in 1991, this generation underwent a profound nutrition transition as adults (18, 19). As this generation ages, the opportunity to study the long-term cardiometabolic consequences of these combined exposures is irreversibly closing, making this a time-sensitive and scientifically unique contribution to understanding the cardiometabolic legacy of the post-Soviet nutrition transition in Central Asia. Furthermore, examining urban–rural differences may provide important evidence regarding how socioeconomic and environmental factors influence the distribution of obesity in Kazakhstan. Therefore, this study aimed to examine the prevalence of overweight, obesity, and central obesity, and to assess socioeconomic inequalities and associated demographic factors among urban and rural older adults in the Astana region of Kazakhstan.

## Materials and methods

### Study population

This cross-sectional study was conducted in the Astana region between November 2012 and March 2015. The urban area was Astana, the capital city of Kazakhstan, and the rural population was the Akmol village, located approximately 50 km from Astana. Participants aged 50–74 years were randomly selected from registries of residents attending local outpatient clinics (polyclinics), with stratification by sex and 5-year age groups.

Eligible participants were initially contacted by landline telephone and invited to attend a single dedicated assessment center in each area, one in Astana and one in Akmol. When telephone contact was unsuccessful, home visits were conducted to invite them to participate. A total of 977 adults were enrolled, including 493 participants from Astana and 484 from Akmol. The overall response rate was 59%, with 56% in urban and 63% in rural areas.

Data were collected by trained physicians using a standardized study protocol. Information was obtained using a structured questionnaire that included sociodemographic characteristics, lifestyle behaviors, medical history, and self-reported health indicators. Clinical examination included blood pressure measurements and anthropometric measurements, including height, weight, waist circumference, and hip circumference. Venous blood samples were collected following an overnight fast. Participants who did not meet fasting requirements were invited to return for repeat assessment. A detailed description of the study design and selected cardiometabolic variables has been published previously (20).

### Measurements

#### Obesity

We used two indices to assess obesity: body mass index (BMI, kg/m^2^) for overweight and overall obesity, and waist-to-hip ratio (WHR) for central obesity (21). Weight was measured to the nearest 0.1 kg using standard balanced scales, with participants wearing light clothing and no shoes. Height was measured to the nearest 0.1 cm using a steel stadiometer.

BMI was calculated as weight in kilograms divided by height in meters squared (kg/m^2^). Following the WHO guidelines, BMI was classified as normal (18.5–24.9), overweight (25–29.9), and obese (BMI > 30 kg/m^2^).

Waist and hip circumferences were measured with a standard tape measure: waist circumference at the midpoint between the costal margin and iliac crest, and hip circumference at the widest part of the gluteal region. WHR was calculated by dividing waist circumference by hip circumference. Based on the WHO-recommended cut-offs, central obesity was defined as WHR > 0.90 in men and > 0.85 in women (21). WHR was selected as the measure of central obesity rather than waist circumference alone because the WHO Expert Consultation (21) recommends both measures as valid indicators of central adiposity, WHR may better reflect body fat distribution in Central Asian populations where standard waist circumference cut-offs derived from Western populations may not be optimally applicable (22), and its use ensures methodological consistency with prior publications from this cohort (20).

#### Cardiometabolic factors

Diabetes was defined as fasting plasma glucose (FPG) > 7.0 mmol/L (126 mg/dL) or self-reported use of antidiabetic medication, without specifying the type of diabetes. Detailed measurement methods and indices were previously described (20). Arterial hypertension was diagnosed after participants rested for 5 min, with blood pressure measured on the right arm using an OMRON M6 Comfort device in a seated position, with three consecutive readings taken at two-minute intervals (23, 24). Hypertension was defined as systolic blood pressure (SBP) ≥ 140 mmHg and/or diastolic blood pressure (DBP) ≥ 90 mmHg, or current use of antihypertensive medication. Hypercholesterolemia was defined as total serum cholesterol ≥6.2 mmol/L (240 mg/dL) or current use of cholesterol-lowering medication, as previously reported (20). Smoking status was assessed using self-reported smoking history. Participants were categorized as current smokers, former smokers, or nonsmokers. Alcohol consumption frequency was assessed using a single item capturing overall drinking frequency in the past 12 months, categorized as never, less than three times per month, and three or more times per month. Although more detailed alcohol quantity and binge-drinking items were collected, these were excluded from analysis due to substantial missingness (approximately 50%) and near-absence of variability among valid responses, consistent with general patterns of alcohol-related stigma and underreporting in self-report research, particularly among older populations (25, 26).

#### Socio-demographic characteristics

Socio-demographic characteristics included age, sex, and place of residence (urban or rural). Marital status was dichotomized into married or unmarried, with the latter including participants who were single, divorced, or widowed. Educational attainment was categorized as primary education or less, vocational/secondary education, or university education. Ethnicity was categorized as Kazakh, Russian, or other ethnic groups. Household asset ownership was used as the primary measure of socioeconomic position, derived from a 14-item possession index including domestic appliances (microwave, television, DVD player, washing machine, dishwasher, freezer, video camera), communication and technology items (cable television, home telephone, mobile telephone, internet access), financial assets (bank account, payment card), and leisure property (dacha). The total possession score was categorized into quartiles (Q1, most deprived to Q4, least deprived).

### Statistical analysis

Descriptive statistics were presented as frequencies and percentages for categorical variables and means with standard deviations (SD) for continuous variables. Differences in participant characteristics between urban and rural residents were assessed using Pearson chi-square tests for categorical variables and independent samples t-tests for continuous variables. Prevalence of overweight, obesity, and central obesity with 95% confidence intervals (CIs) was stratified by sociodemographic characteristics. Differences in prevalence across subgroups were assessed using Pearson chi-square tests.

Binary logistic regression was used to estimate odds ratios (ORs) and 95% CIs for three binary outcomes: overweight (BMI ≥ 25 kg/m^2^), obesity (BMI ≥ 30 kg/m^2^), and central obesity (WHR > 0.90 in men and >0.85 in women). Three sequential models were fitted for each outcome. Model 1 was an age- and sex-adjusted model, with each exposure variable estimated in a separate binary logistic regression. Model 2 was additionally adjusted for cardiometabolic and behavioral variables (diabetes, hypertension, hypercholesterolemia, smoking status, and alcohol consumption frequency), with each sociodemographic variable estimated from a separate model. Model 3 was the fully adjusted model, including all sociodemographic, behavioral, and cardiometabolic variables simultaneously. Diabetes, hypertension, and hypercholesterolemia were included as covariates in Models 2 and 3 because, in this older adult population, these conditions are likely longstanding and may predate current obesity rather than arising solely from it. We acknowledge that in a cross-sectional study, temporal ordering cannot be established with certainty, and these variables may act as partial mediators for some participants. A sensitivity analysis excluding these three variables was therefore conducted to assess the robustness of the socioeconomic gradient estimates (Supplementary Table 4). Robust standard errors were used in all models to account for potential heteroskedasticity and to provide valid inference under possible misspecification of the variance structure. Regarding sampling design: polyclinic registries served as the administrative sampling frame from which participants were randomly selected, with stratification by sex and 5-year age group applied within each site. Participants were not recruited through or nested within individual polyclinics as distinct clinical units, and no within-cluster correlation structure was induced by the sampling procedure. Urban and rural residence were explicitly included as a covariate in all multivariable models to account for site-level differences between Astana and Akmol. Under these conditions, robust standard errors provide a conservative and appropriate approach to variance estimation without the need for formal cluster-adjusted or survey-weighted methods.

Prevalence estimates were calculated among all participants with valid data for each variable (*n* = 969 for BMI outcomes; *n* = 964 for central obesity). Multivariable regression analyses were restricted to participants with complete data on all covariates included in the fully adjusted model (*n* = 854 for overweight and obesity; *n* = 852 for central obesity). Prior to multivariable modeling, multicollinearity among covariates was assessed by computing variance inflation factors (VIF) using an equivalent ordinary least squares regression; all VIF values ranged from 1.05 to 2.07 (mean VIF 1.44), indicating no problematic multicollinearity. Model fit was assessed using the Hosmer-Lemeshow goodness-of-fit test with 10 groups, and discriminative ability was evaluated using the area under the receiver operating characteristic curve (AUC). Results are reported in Supplementary Table 2.

Three sensitivity analyses were conducted. First, to assess potential bias from missing data, participants included in the fully adjusted models were compared with those excluded due to missing covariate data on key outcome and demographic variables using Pearson chi-square tests; results are reported in Supplementary Table 1. Second, to formally test for a linear dose–response relationship between household asset ownership and obesity outcomes, household asset quartile was additionally modeled as a continuous variable in the fully adjusted model, with the resulting coefficient interpreted as the odds ratio per one-quartile increase in asset ownership; results are reported in Supplementary Table 3. Third, although physical activity, occupational history, and detailed dietary data were not available for the present analysis, alcohol consumption frequency was included as a behavioral covariate in Models 2 and 3 to partially address potential behavioral confounding. The dose–response relationship between household asset ownership and the predicted probability of obesity outcomes was visualized using marginal effects plots derived from the fully adjusted model, with household asset quartiles treated as continuous variables (Supplementary Figure 1). All analyses were conducted using STATA version 17 (College Station, Texas, USA).

## Results

The descriptive characteristics of the study sample stratified by place of residence are presented in Table 1. The analytic sample comprised 493 urban (Astana) and 484 rural (Akmol) participants, with a higher proportion of women in both settings (53.3% urban, 58.3% rural). Age and sex distributions did not differ significantly between urban and rural participants (*p* = 0.170 and *p* = 0.122, respectively). Ethnic Kazakhs comprised 58.1% of participants, with Russians (26.4%) and other ethnicities (15.5%) making up the remainder; ethnic composition did not differ by residence (*p* = 0.765). Married participants accounted for 73.5% of the sample, with no significant difference between urban and rural residents (*p* = 0.823).

**Table 1:** Sociodemographic and clinical characteristics of study participants by residence, Astana region, Kazakhstan.

Characteristic	Urban (Astana) *N* = 493	Rural (Akmol) *N* = 484	Total *N* = 977	*p*-value
Sex, *n* (%)				0.122
Male	230 (46.7)	202 (41.7)	432 (44.2)	
Female	263 (53.3)	282 (58.3)	545 (55.8)	
Age group, *n* (%)				0.170
50–54 years	126 (25.6)	138 (28.5)	264 (27.0)	
55–59 years	101 (20.5)	123 (25.4)	224 (22.9)	
60–64 years	109 (22.1)	92 (19.0)	201 (20.6)	
65–69 years	80 (16.2)	66 (13.6)	146 (14.9)	
70–74 years	77 (15.6)	65 (13.4)	142 (14.5)	
Weight (kg), mean (SD)	79.41 (14.69)	76.65 (14.56)	78.04 (14.69)	0.003
Height (cm), mean (SD)	163.30 (8.66)	162.09 (8.71)	162.70 (8.70)	0.031
BMI (kg/m^2^), mean (SD)	29.77 (4.94)	29.21 (5.30)	29.49 (5.13)	0.090
BMI category, n (%)				0.049
Normal weight	78 (16.0)	107 (22.2)	185 (19.1)	
Overweight	186 (38.2)	173 (35.9)	359 (37.1)	
Obesity	223 (45.8)	202 (41.9)	425 (43.9)	
Waist circumference (cm), mean (SD)	98.03 (11.80)	95.01 (13.55)	96.52 (12.79)	<0.001
Hip circumference (cm), mean (SD)	106.20 (9.55)	105.60 (11.04)	105.90 (10.32)	0.366
Waist-to-hip ratio, mean (SD)	0.924 (0.088)	0.901 (0.106)	0.913 (0.098)	<0.001
Central obesity, *n* (%)				<0.001
Normal WHR	89 (18.5)	184 (38.2)	273 (28.3)	
High WHR	393 (81.5)	298 (61.8)	691 (71.7)	
Diabetes, *n* (%)				<0.001
No	400 (83.7)	434 (91.4)	834 (87.5)	
Yes	78 (16.3)	41 (8.6)	119 (12.5)	
Hypertension, *n* (%)				0.297
No	144 (29.4)	125 (26.4)	269 (27.9)	
Yes	346 (70.6)	349 (73.6)	695 (72.1)	
Hypercholesterolemia, *n* (%)				<0.001
No	248 (51.6)	352 (74.3)	600 (62.8)	
Yes	233 (48.4)	122 (25.7)	355 (37.2)	
Smoking status, *n* (%)				0.016
Non-smoker	294 (60.6)	332 (69.5)	626 (65.0)	
Current smoker	86 (17.7)	66 (13.8)	152 (15.8)	
Past smoker	105 (21.6)	80 (16.7)	185 (19.2)	
Alcohol consumption, *n* (%)				<0.001
Never	194 (40.3)	292 (62.1)	486 (51.1)	
<3 times/month	251 (52.2)	168 (35.7)	419 (44.1)	
≥3 times/month	36 (7.5)	10 (2.1)	46 (4.8)	
Marital status, *n* (%)				0.823
Married	360 (73.2)	355 (73.8)	715 (73.5)	
Unmarried	132 (26.8)	126 (26.2)	258 (26.5)	
Education, *n* (%)				<0.001
University	215 (43.6)	73 (15.2)	288 (29.6)	
Vocational/secondary	149 (30.2)	183 (38.1)	332 (34.1)	
Primary or less	129 (26.2)	224 (46.7)	353 (36.3)	
Ethnicity, *n* (%)				0.765
Kazakh	283 (57.8)	283 (58.5)	566 (58.1)	
Russian	127 (25.9)	130 (26.9)	257 (26.4)	
Other	80 (16.3)	71 (14.7)	151 (15.5)	
Household possessions, *n* (%)				<0.001
Q1 Most deprived	78 (16.8)	152 (32.7)	230 (24.8)	
Q2 Partially deprived	99 (21.3)	170 (36.6)	269 (29.0)	
Q3 Moderately deprived	162 (34.9)	129 (27.7)	291 (31.3)	
Q4 Least deprived	125 (26.9)	14 (3.0)	139 (15.0)	

Values are *N* (%) or mean (SD). BMI, body mass index; WHR, waist-to-hip ratio; SD, standard deviation. Normal weight: BMI 18.5–24.9 kg/m^2^; Overweight: BMI 25.0–29.9 kg/m^2^; Obesity: BMI ≥30.0 kg/m^2^; Central obesity: WHR >0.90 in men and >0.85 in women. *p* - values from Pearson chi-square tests for categorical variables and independent samples t-tests for continuous variables. *p* < 0.05 was considered statistically significant.

Urban and rural participants differed significantly on several key characteristics. Urban residents had substantially higher educational attainment (43.6% university vs. 15.2%, *p* < 0.001), greater household asset ownership (26.9% in the highest quartile vs. 3.0%, *p* < 0.001), higher rates of hypercholesterolemia (48.4% vs. 25.7%, *p* < 0.001), and higher diabetes prevalence (16.3% vs. 8.6%, *p* < 0.001). Urban participants also reported higher rates of current and past smoking and greater alcohol consumption (both *p* < 0.05). Mean waist circumference (98.03 vs. 95.01 cm, *p* < 0.001) and waist-to-hip ratio (0.924 vs. 0.901, *p* < 0.001) were significantly higher in urban than rural participants. BMI did not differ significantly between urban and rural residents (29.77 vs. 29.21 kg/m^2^, *p* = 0.090).

The prevalence of overweight, obesity, and central obesity by sociodemographic characteristics is presented in Table 2. The overall prevalence of overweight was 37.1% (95% CI, 34.1–40.1%), obesity was 43.9% (95% CI, 40.8–47.0%), and central obesity was 71.7% (95% CI, 68.8–74.4%). Obesity prevalence was significantly higher in women than in men (51.7% vs. 33.9%, *p* < 0.001), while central obesity was more prevalent in men than in women (78.9% vs. 66.1%, p < 0.001). Central obesity prevalence increased significantly with age (*p* = 0.001), rising from 65.4% in the 50–54 age group to 83.6% in the 70–74 age group, while no significant age gradient was observed for BMI-based overweight or obesity (*p* = 0.209). Urban residents had a substantially higher prevalence of central obesity than rural residents (81.5% vs. 61.8%, *p* < 0.001), while BMI category distribution showed only a borderline significant difference by residence (*p* = 0.049). Obesity prevalence increased with household asset ownership (Q1: 33.3% to Q3: 49.3%, *p* = 0.002), and central obesity showed a similar pattern (Q1: 67.0% to Q4: 84.2%, *p* = 0.001). Ethnicity was significantly associated with BMI category (*p* < 0.001) but not with central obesity (*p* = 0.477), with non-Kazakh ethnic groups showing higher obesity prevalence than Kazakhs.

**Table 2:** Prevalence of overweight, obesity, and central obesity by sociodemographic characteristics among older adults in the Astana region of Kazakhstan.

Characteristic	Category	Normal Weight % (95% CI)	Overweight % (95% CI)	Obesity % (95% CI)	*p*-value*	Central Obesity % (95% CI)	*p*-value**
Overall		19.09 (16.74–21.69)	37.05 (34.06–40.14)	43.86 (40.76–47.01)		71.68 (68.75–74.43)	
Sex					<0.001		<0.001
	Male	24.47 (20.61–28.80)	41.65 (37.04–46.41)	33.88 (29.53–38.53)		78.86 (74.68–82.51)	
	Female	14.89 (12.13–18.14)	33.46 (29.61–37.54)	51.65 (47.44–55.84)		66.11 (62.01–69.99)	
Age group (years)					0.209		0.001
	50–54	20.53 (16.06–25.87)	32.70 (27.28–38.62)	46.77 (40.79–52.84)		65.40 (59.42–70.93)	
	55–59	17.65 (13.14–23.28)	40.27 (33.97–47.09)	42.08 (35.71–48.73)		67.87 (61.40–73.73)	
	60–64	23.74 (18.29–30.20)	34.85 (28.50–41.79)	41.41 (34.72–48.44)		75.00 (68.42–80.60)	
	65–69	15.86 (10.74–22.81)	35.86 (28.22–44.41)	48.28 (40.19–56.45)		72.92 (65.02–79.59)	
	70–74	15.49 (10.39–22.48)	44.37 (36.35–52.69)	40.14 (32.34–48.47)		83.57 (76.42–88.88)	
Residence					0.117		<0.001
	Urban (Astana)	16.02 (13.01–19.56)	38.19 (33.97–42.60)	45.79 (41.40–50.25)		81.54 (77.81–84.76)	
	Rural (Akmol)	22.20 (18.70–26.14)	35.89 (31.72–40.29)	41.91 (37.57–46.38)		61.83 (57.39–66.07)	
Marital status					0.244		0.078
	Married	20.48 (17.66–23.62)	36.30 (32.83–39.91)	43.22 (39.61–46.91)		73.15 (69.75–76.30)	
	Unmarried	15.56 (11.61–20.55)	39.30 (33.49–45.43)	45.14 (39.13–51.29)		67.19 (61.18–72.69)	
Education					0.722		0.003
	University	17.96 (13.90–22.88)	41.20 (35.59–47.04)	40.85 (35.25–46.69)		78.45 (73.24–82.87)	
	Vocational/secondary	19.39 (15.47–24.04)	35.76 (30.75–41.10)	44.85 (39.54–50.27)		64.94 (59.60–69.93)	
	Primary or less	19.66 (15.82–24.17)	34.76 (29.94–39.91)	45.58 (40.42–50.84)		72.49 (67.55–76.94)	
Ethnicity					<0.001		0.477
	Kazakh	23.49 (20.16–27.18)	37.72 (33.80–41.81)	38.79 (34.84–42.90)		72.78 (68.93–76.30)	
	Russian	12.60 (9.04–17.30)	38.58 (32.77–44.74)	48.82 (42.69–54.98)		68.40 (62.35–73.89)	
	Other	13.33 (8.74–19.83)	32.67 (25.59–40.63)	54.00 (45.92–61.87)		73.15 (65.41–79.70)	
Household possessions					0.002		0.001
	Q1 Most deprived	28.07 (22.5–34.29)	38.60 (32.47–45.11)	33.33 (27.49–39.74)		66.96 (60.55–72.80)	
	Q2 Partially deprived	17.74 (13.58–22.83)	36.98 (31.35–42.98)	45.28 (39.36–51.34)		67.05 (61.12–72.47)	
	Q3 Moderately deprived	15.52 (11.78–20.18)	35.17 (29.87–40.87)	49.31 (43.57–55.07)		72.32 (66.85–77.19)	
	Q4 Least deprived	14.39 (9.44–21.32)	39.57 (31.72–47.99)	46.04 (37.87–54.40)		84.17 (77.06–89.38)	

Values are prevalence % (95% confidence interval). BMI, body mass index; WHR, waist-to-hip ratio. Normal weight: BMI 18.5–24.9 kg/m^2^; Overweight: BMI 25.0–29.9 kg/m^2^; Obesity: BMI ≥30.0 kg/m^2^; Central obesity: WHR >0.90 in men and >0.85 in women. *p-value from Pearson chi-square test for difference in BMI category distribution across subgroups. **p-value from Pearson chi-square test for difference in central obesity prevalence across subgroups. *p* < 0.05 was considered statistically significant.

The associations between sociodemographic, behavioral, and cardiometabolic factors and the three obesity outcomes across all three models are presented in Table 3.

**Table 3:** Binary logistic regression analyses of factors associated with overweight, obesity, and central obesity among older adults in the Astana region of Kazakhstan.

Variable	Category	Overweight	Overweight	Overweight	Obesity	Obesity	Obesity	Central obesity	Central obesity	Central obesity
		OR^1^ (95% CI)	OR^2^ (95% CI)	OR^3^ (95% CI)	OR^1^ (95% CI)	OR^2^ (95% CI)	OR^3^ (95% CI)	OR^1^ (95% CI)	OR^2^ (95% CI)	OR^3^ (95% CI)
Sex	Male (ref)	1.00	1.00	1.00	1.00	1.00	1.00	1.00	1.00	1.00
	Female	1.84 (1.33–2.54)***	1.48 (0.96–2.29)	1.28 (0.79–2.09)	2.10 (1.62–2.74)***	2.35 (1.59–3.49)***	2.55 (1.65–3.94)***	0.52 (0.39–0.71)***	0.55 (0.36–0.84)**	0.68 (0.43–1.06)
Age group (years)	50–54 (ref)	1.00	1.00	1.00	1.00	1.00	1.00	1.00	1.00	1.00
	55–59	1.16 (0.73–1.85)	1.08 (0.65–1.80)	1.10 (0.64–1.89)	0.78 (0.54–1.14)	0.71 (0.48–1.06)	0.61 (0.40–0.93)*	1.17 (0.80–1.71)	1.07 (0.71–1.63)	1.26 (0.80–1.98)
	60–64	0.85 (0.54–1.33)	0.73 (0.44–1.19)	0.67 (0.40–1.13)	0.82 (0.56–1.20)	0.74 (0.49–1.12)	0.67 (0.43–1.04)	1.57 (1.04–2.37)*	1.27 (0.81–2.00)	1.28 (0.80–2.05)
	65–69	1.38 (0.81–2.36)	1.34 (0.74–2.45)	1.13 (0.60–2.12)	1.07 (0.71–1.61)	1.01 (0.64–1.59)	0.90 (0.55–1.47)	1.44 (0.91–2.25)	1.17 (0.72–1.91)	1.12 (0.67–1.87)
	70–74	1.41 (0.82–2.44)	1.09 (0.59–2.02)	0.96 (0.48–1.93)	0.76 (0.50–1.15)	0.57 (0.36–0.91)*	0.54 (0.33–0.90)*	2.74 (1.62–4.64)***	1.98 (1.10–3.56)*	2.30 (1.19–4.42)*
Diabetes	No (ref)	1.00	1.00	1.00	1.00	1.00	1.00	1.00	1.00	1.00
	Yes	4.32 (2.00–9.33)***	3.44 (1.58–7.50) **	2.99 (1.37–6.52)**	2.97 (1.96–4.50)***	3.02 (1.94–4.68)***	2.84 (1.80–4.48)***	5.38 (2.64–10.97)***	4.61 (2.22–9.58)***	5.04 (2.29–11.10)***
Hypertension	No (ref)	1.00	1.00	1.00	1.00	1.00	1.00	1.00	1.00	1.00
	Yes	2.54 (1.81–3.58)***	2.35 (1.63–3.40)***	2.29 (1.56–3.37)***	2.29 (1.68–3.12)***	2.20 (1.59–3.05)***	2.25 (1.58–3.19)***	1.93 (1.41–2.65)***	1.69 (1.21–2.37)**	1.77 (1.23–2.54)**
High cholesterol	No (ref)	1.00	1.00	1.00	1.00	1.00	1.00	1.00	1.00	1.00
	Yes	1.86 (1.28–2.69)**	1.49 (1.00–2.22)*	1.49 (0.97–2.27)	1.34 (1.02–1.76)*	1.17 (0.87–1.57)	1.13 (0.82–1.56)	2.09 (1.53–2.86)***	1.84 (1.32–2.56)***	1.73 (1.21–2.48)**
Smoking status	Non-smoker (ref)	1.00	1.00	1.00	1.00	1.00	1.00	1.00	1.00	1.00
	Current smoker	0.43 (0.27–0.70) **	0.45 (0.27–0.75)**	0.36 (0.20–0.62)***	0.83 (0.53–1.29)	0.99 (0.61–1.60)	0.96 (0.58–1.60)	0.96 (0.59–1.56)	1.07 (0.65–1.77)	1.20 (0.71–2.04)
	Past smoker	1.16 (0.69–1.94)	1.05 (0.61–1.81)	0.89 (0.50–1.57)	1.54 (0.99–2.38)	1.63 (1.02–2.59)*	1.57 (0.96–2.57)	1.63 (0.96–2.75)	1.53 (0.89–2.64)	1.64 (0.94–2.87)
Alcohol consumption	Never (ref)	1.00	1.00	1.00	1.00	1.00	1.00	1.00	1.00	1.00
	<3 times/month	1.55 (1.10–2.20) *	1.62 (1.11–2.36)*	1.32 (0.87–2.01)	1.00 (0.76–1.31)	0.94 (0.70–1.26)	0.80 (0.58–1.10)	1.11 (0.82–1.50)	0.98 (0.72–1.35)	0.94 (0.66–1.35)
	≥3 times/month	2.25 (0.97–5.23)	2.32 (0.90–6.03)	1.77 (0.66–4.72)	1.30 (0.69–2.44)	1.04 (0.48–2.15)	0.81 (0.37–1.77)	2.08 (0.86–5.05)	1.86 (0.70–4.97)	1.25 (0.43–3.63)
Residence	Urban (ref)	1.00	1.00	1.00	1.00	1.00	1.00	1.00	1.00	1.00
	Rural	0.64 (0.46–0.89)**	0.63 (0.44–0.92)*	0.67 (0.43–1.04)	0.82 (0.63–1.06)	0.88 (0.65–1.18)	0.85 (0.60–1.18)	0.38 (0.28–0.51)***	0.40 (0.28–0.56)***	0.43 (0.30–0.64)***
Marital status	Not married (ref)	1.00	1.00	1.00	1.00	1.00	1.00	1.00	1.00	1.00
	Married	1.04 (0.67–1.61)	1.25 (0.78–2.00)	1.30 (0.79–2.15)	0.78 (0.57–1.07)	0.78 (0.55–1.11)	0.74 (0.52–1.07)	0.85 (0.60–1.20)	0.82 (0.56–1.18)	0.75 (0.51–1.12)
Education	University (ref)	1.00	1.00	1.00	1.00	1.00	1.00	1.00	1.00	1.00
	Secondary/vocational	0.82 (0.53–1.26)	0.99 (0.62–1.59)	1.00 (0.59–1.68)	1.06 (0.76–1.49)	1.14 (0.80–1.62)	1.20 (0.81–1.78)	0.55 (0.38–0.80)**	0.62 (0.42–0.91)*	0.81 (0.52–1.25)
	Primary or less	0.83 (0.54–1.26)	1.06 (0.68–1.67)	1.25 (0.74–2.09)	1.19 (0.86–1.65)	1.32 (0.93–1.89)	1.56 (1.03–2.34)*	0.67 (0.45–0.98)*	0.71 (0.47–1.07)	1.04 (0.65–1.66)
Ethnicity	Kazakh (ref)	1.00	1.00	1.00	1.00	1.00	1.00	1.00	1.00	1.00
	Russian	1.95 (1.27–2.98)**	1.86 (1.15–3.00)*	2.05 (1.23–3.42)**	1.45 (1.07–1.97)*	1.37 (0.97–1.93)	1.46 (1.02–2.08)*	0.80 (0.57–1.12)	0.63 (0.43–0.93)*	0.66 (0.44–0.99)*
	Other	1.86 (1.11–3.11)*	2.09 (1.16–3.79)*	2.43 (1.28–4.59)**	1.85 (1.27–2.70)**	1.86 (1.25–2.76)**	1.89 (1.25–2.87)**	0.99 (0.65–1.50)	0.82 (0.52–1.30)	0.98 (0.58–1.64)
Household assets	Q1 Most deprived	1.00	1.00	1.00	1.00	1.00	1.00	1.00	1.00	1.00
	Q2 Partially deprived	1.88 (1.21–2.91)**	1.36 (0.85–2.18)	1.31 (0.81–2.12)	1.62 (1.12–2.35)*	1.30 (0.87–1.95)	1.36 (0.90–2.04)	1.08 (0.73–1.59)	0.88 (0.58–1.33)	0.85 (0.55–1.31)
	Q3 Moderately deprived	2.42 (1.53–3.83)***	1.76 (1.08–2.89)*	1.68 (1.00–2.80)*	2.08 (1.44–3.01)***	1.94 (1.30–2.90)**	2.05 (1.35–3.11)**	1.42 (0.96–2.09)	1.18 (0.78–1.80)	0.97 (0.62–1.54)
	Q4 Least deprived	2.96 (1.63–5.38)***	1.97 (1.02–3.80)*	1.84 (0.90–3.77)	2.01 (1.27–3.17)**	1.66 (1.02–2.70)*	1.88 (1.09–3.25)*	2.87 (1.65–5.00)***	2.10 (1.17–3.78)*	1.22 (0.63–2.36)

OR, odds ratio; CI, confidence interval; BMI, body mass index; WHR, waist-to-hip ratio. OR^1^: age- and sex-adjusted odds ratio, with each variable estimated from a separate binary logistic regression model. OR^2^: additionally adjusted for diabetes, hypertension, hypercholesterolemia, smoking status, and alcohol consumption frequency, with each sociodemographic variable estimated from a separate model. OR^3^: fully adjusted odds ratio from a single binary logistic regression model including all variables simultaneously (overweight and obesity: *n* = 854; central obesity: *n* = 852). Robust standard errors were used throughout. *p < 0.05; **p < 0.01; ***p < 0.001.

In the age- and sex-adjusted model (Model 1), women had significantly higher odds of overweight (OR 1.84, 95% CI 1.33–2.54) and obesity (OR 2.10, 95% CI 1.62–2.74) compared with men, while women had significantly lower odds of central obesity (OR 0.52, 95% CI 0.39–0.71). In the fully adjusted model (Model 3), the association between female sex and overweight was no longer statistically significant (OR 1.28, 95% CI 0.79–2.09), while the higher odds of obesity in women persisted (OR 2.55, 95% CI 1.65–3.94). The sex difference in central obesity was attenuated and no longer significant in Model 3 (OR 0.68, 95% CI 0.43–1.06). No significant age gradient was observed for overweight or obesity across any model. For central obesity, participants aged 70–74 years had significantly greater odds than the youngest age group in all three models (Model 3: OR 2.30, 95% CI 1.19–4.42).

Diabetes, hypertension, and hypercholesterolemia were consistently and significantly associated with all three obesity outcomes across all models. In the fully adjusted model, diabetes was most strongly associated with central obesity (OR 5.04, 95% CI 2.29–11.10), followed by overweight (OR 2.99, 95% CI 1.37–6.52) and obesity (OR 2.84, 95% CI 1.80–4.48). Hypertension showed consistent associations across all three outcomes in Model 3 (overweight: OR 2.29, 95% CI 1.56–3.37; obesity: OR 2.25, 95% CI 1.58–3.19; central obesity: OR 1.77, 95% CI 1.23–2.54). Hypercholesterolemia was significantly associated with central obesity in all models (Model 3: OR 1.73, 95% CI 1.21–2.48) but lost statistical significance for overweight and obesity after full adjustment.

Current smokers had significantly lower odds of being overweight compared with non-smokers across all three models (Model 3: OR 0.36, 95% CI 0.20–0.62), while no significant association was observed for obesity or central obesity. Past smokers showed no significant association with any outcome in any model. Alcohol consumption frequency was not significantly associated with any of the three obesity outcomes in any model, though participants consuming alcohol less than three times per month showed a modest positive association with overweight in Models 1 and 2 that was attenuated in the fully adjusted model.

Rural residence was significantly associated with lower odds of overweight in Models 1 and 2, but this association was attenuated to borderline significance in Model 3 (OR 0.67, 95% CI 0.43–1.04, *p* = 0.073). For obesity, rural residents showed no significant association in any model. In contrast, rural residence was strongly and consistently protective against central obesity across all three models (Model 1: OR 0.38, 95% CI 0.28–0.51; Model 3: OR 0.43, 95% CI 0.30–0.64).

Marital status was not significantly associated with any obesity outcome in any model. Educational attainment was not significantly associated with overweight in any model. For obesity, participants with primary or less education had significantly higher odds compared with university-educated participants in the fully adjusted model (OR 1.56, 95% CI 1.03–2.34), while no significant association was observed at the secondary/vocational level. For central obesity, education showed a significant inverse association in Model 1 (secondary/vocational: OR 0.55, 95% CI 0.38–0.80; primary or less: OR 0.67, 95% CI 0.45–0.98) that was fully attenuated in Model 3, suggesting that this association was confounded by other variables in the fully adjusted model.

Compared with Kazakhs, Russians and other ethnic groups had significantly higher odds of overweight and obesity across all three models. In the fully adjusted model, Russian ethnicity was associated with twice the odds of overweight (OR 2.05, 95% CI 1.23–3.42) and significantly higher odds of obesity (OR 1.46, 95% CI 1.02–2.08). Other ethnic groups showed similarly elevated odds for both overweight (OR 2.43, 95% CI 1.28–4.59) and obesity (OR 1.89, 95% CI 1.25–2.87). In contrast, for central obesity, Russian ethnicity was associated with significantly lower odds compared with Kazakhs (Model 3: OR 0.66, 95% CI 0.44–0.99), representing an opposite directional pattern to that observed for BMI-based outcomes.

A consistent positive association between household asset ownership and BMI-based obesity outcomes was observed across all models, consistent with a reversed socioeconomic gradient. In the age- and sex-adjusted model, participants in the highest asset quartile (least deprived) had nearly three times the odds of overweight (OR 2.96, 95% CI 1.63–5.38) and twice the odds of obesity (OR 2.01, 95% CI 1.27–3.17) compared with those in the most deprived quartile. These associations were attenuated after adjustment for cardiometabolic conditions and behavioral variables in Model 2 but remained statistically significant. In the fully adjusted model, the third asset quartile remained significantly associated with both overweight (OR 1.68, 95% CI 1.00–2.80) and obesity (OR 2.05, 95% CI 1.35–3.11), and the highest quartile remained significantly associated with obesity (OR 1.88, 95% CI 1.09–3.25). A formal test for linear trend across asset quartiles confirmed a statistically significant positive dose–response relationship for overweight (OR per quartile increase 1.25, 95% CI 1.01–1.55, p for trend = 0.038) and obesity (OR per quartile increase 1.30, 95% CI 1.10–1.54, p for trend = 0.002). For central obesity, household asset ownership showed a significant positive association in Models 1 and 2 but was fully attenuated in the fully adjusted model, with no significant linear trend observed (OR per quartile increase 1.04, 95% CI 0.86–1.25, p for trend = 0.685), indicating that the reversed socioeconomic gradient is specific to BMI-based obesity outcomes. The dose–response relationship is further illustrated in Supplementary Figure 1, which shows predicted probabilities of overweight, obesity, and central obesity across household asset quartiles from the fully adjusted model. Predicted probabilities of overweight and obesity increased progressively from Q1 (most deprived) to Q4 (least deprived), while predicted probability of central obesity remained relatively stable across quartiles, visually confirming the outcome-specific nature of the reversed socioeconomic gradient. A sensitivity analysis excluding diabetes, hypertension, and hypercholesterolemia confirmed that the reversed socioeconomic gradient for overweight and obesity remained consistent in direction, magnitude, and statistical significance (overweight Q4: OR 2.28, 95% CI 1.15–4.49; obesity Q4: OR 1.96, 95% CI 1.16–3.30), with point estimates changing by less than 25% compared with the primary models, indicating no meaningful overadjustment. The association between household asset ownership and central obesity remained non-significant, consistent with the primary findings. Full results are presented in Supplementary Table 4.

Three additional sensitivity analyses were conducted to address methodological concerns. First, to assess potential selection bias from missing covariate data, participants included in the fully adjusted models were compared with those excluded on key sociodemographic, socioeconomic, and outcome characteristics. No significant differences were found between included and excluded participants on education, household asset quartile, ethnicity, place of residence, outcome category, sex, or age group in either analytic sample (all *p* > 0.05), indicating that listwise deletion was unlikely to introduce systematic bias (Supplementary Table 1). Second, model fit and discriminative ability were assessed for all three fully adjusted models: Hosmer-Lemeshow goodness-of-fit tests showed no significant lack of fit for overweight (χ^2^ = 8.81, df = 8, *p* = 0.359), obesity (χ^2^ = 4.51, df = 8, *p* = 0.808), or central obesity (χ^2^ = 6.13, df = 8, *p* = 0.633), and areas under the ROC curve were 0.747, 0.712, and 0.740 respectively, indicating acceptable discriminative ability (Supplementary Table 2). Third, to formally assess the dose–response nature of the socioeconomic gradient, household asset quartile was modeled as a continuous variable; a significant positive linear trend was confirmed for overweight (p for trend = 0.038) and obesity (p for trend = 0.002) but not for central obesity (p for trend = 0.685), supporting a genuine dose–response reversed gradient specific to BMI-based outcomes (Supplementary Table 3). Prior to multivariable modeling, variance inflation factors ranged from 1.05 to 2.07 (mean VIF 1.44), indicating no problematic multicollinearity among covariates.

## Discussion

In this population-based study of older adults in the Astana region of Kazakhstan, the prevalence of overweight, obesity, and central obesity was notably high. Overall, 37% of participants were overweight, 44% were obese, and 72% had central obesity. Men demonstrated a higher prevalence of overweight and central obesity, whereas BMI-defined obesity was more common among women; 52% of women and 34% of men were obese, while 42% of men and 34% of women were overweight. Overweight, obesity, and central obesity were all significantly and consistently associated with diabetes, hypertension, and hypercholesterolemia across all models. Higher odds of overweight and obesity were observed among individuals with greater household asset ownership, suggesting a reversed socioeconomic gradient consistent with an early-stage nutrition transition. By contrast, lower educational attainment was associated with higher odds of obesity in the fully adjusted model, reflecting a more complex and divergent pattern between these two socioeconomic indicators. In addition, Russian and other non-Kazakh ethnic groups had higher odds of overweight and obesity compared with ethnic Kazakhs. Older age was consistently associated with higher odds of central obesity. These findings highlight the substantial burden of obesity and cardiometabolic conditions among older adults in Kazakhstan and reveal important socioeconomic and demographic disparities in obesity distribution.

Population-based research examining socioeconomic inequalities in overweight and obesity in Central Asia remains limited (27). This study contributes to the growing evidence base by providing data on overweight, obesity, and central obesity among older adults in Kazakhstan (5, 20). A recent cross-sectional study of 4,800 adults aged 18–69 years in West Kazakhstan reported an overall obesity prevalence of 22.3% (28); however, that study did not examine socioeconomic correlates of obesity and relied on a broader working-age population. The substantially higher obesity prevalence observed in our study (43.9%) is consistent with the well-established increase in obesity risk with age, and our findings extend the regional evidence base by providing the first individual-level analysis of socioeconomic patterning of obesity among older adults in Kazakhstan using objectively measured indicators.

To our knowledge, few studies in the region have examined the socioeconomic and demographic correlates of obesity using objectively measured anthropometric indicators and internationally comparable measurement protocols (5, 20, 22). Critically, the study population represents the last generation of Kazakhstani adults whose entire formative years were shaped by Soviet-era nutritional and socioeconomic conditions, characterized by centralized food distribution, constrained dietary variety, enforced physical labor, and relative socioeconomic homogeneity. Following the dissolution of the USSR in 1991, this cohort underwent one of the most dramatic nutritional and economic transitions in modern public health history as adults (18, 19). As this generation ages, the opportunity to study the long-term cardiometabolic consequences of this lived nutrition transition at the individual level is irreversibly closing, making these findings a scientifically unique and time-sensitive contribution to understanding obesity in this transitional setting.

Sex differences in the prevalence of overweight and obesity have been reported globally, although the direction and magnitude of these differences vary across regions and economic settings. Studies from Western Europe and North America have shown mixed patterns, with obesity prevalence often being similar or slightly higher among women (29, 30). In Eastern Europe and Russia, previous studies have also reported a high prevalence of overweight and obesity, particularly among older adults (14, 19). Findings from the HAPIEE Study conducted in Russia, the Czech Republic, and Poland demonstrated high obesity prevalence among women, ranging from 21% in Russia to 30% in the Czech Republic in men and from 32% in the Czech Republic to 47% in Russia in women; in addition, more than 70% of that study population was overweight (BMI over 25) (14). In our study, in a similar age group, 52% of women and 34% of men were obese, while 42% of men and 34% of women were overweight, findings broadly consistent with this regional literature, though at the higher end of the range for women. Internationally, the gender differences in the prevalence of overweight and obesity are generally higher in lower-income countries, and the gap usually increases with decreasing levels of per capita income (31), which might explain the gender gaps in Central Asia, particularly in Kazakhstan. The risk of obesity increases with age in both men and women across all settings (32), though in our study, no significant age gradient was observed for BMI-based outcomes, possibly reflecting a plateau effect in this already high-risk older-adult cohort.

There are numerous studies on the levels and distribution of obesity in urban and rural regions, and the data vary considerably between populations. Obesity has been reported to be markedly higher among adults in rural than in urban areas in the United States (33), whereas no differences in the prevalence of overweight and obesity were found between rural and urban areas across 10 European countries (34). A more recent study across 20 European countries found that rural residents were significantly more overweight and obese than those living in urban areas (35). In our study, urban and rural participants had broadly comparable BMI distributions, though this difference reached nominal statistical significance (*p* = 0.049), and differed substantially in central obesity prevalence (81.5% urban vs. 61.8% rural, *p* < 0.001), hypercholesterolemia (48.4% vs. 25.7%, *p* < 0.001), and diabetes (16.3% vs. 8.6%, *p* < 0.001), suggesting that urbanization in Kazakhstan is associated with cardiometabolic risk that extends beyond overall adiposity. Rapid urbanization, the introduction of a Western lifestyle, and economic transformation in Kazakhstan may be associated with an accelerated nutrition transition, as observed in other populations undergoing similar socioeconomic change (3).

Abdominal obesity has a central role in the development of a pro-inflammatory state, which is associated with metabolic syndrome (36). A higher prevalence of central obesity worldwide was observed among older subjects, females, urban residents, and people from higher-income countries (37). In our study, central obesity was significantly more prevalent in urban than rural residents and increased progressively with age, reaching 83.6% in the 70–74 age group. Contrary to patterns reported in some settings, men in our study had a higher prevalence of central obesity than women (78.9% vs. 66.1%), though this sex difference was attenuated in the fully adjusted model. In the multivariable analysis, the association with cardiometabolic risk factors, particularly diabetes, became even stronger (OR 5.04, 95% CI 2.29–11.10). Rural residence was the most robust and consistent protective factor against central obesity across all three models (fully adjusted OR 0.43, 95% CI 0.30–0.64), persisting after adjustment for all sociodemographic and cardiometabolic covariates. While an accelerated nutrition transition may account for the high rates of central obesity in this region, other important factors such as increases in physical inactivity and sedentary behavior should also be carefully investigated in low- and middle-income countries undergoing rapid urbanization (38).

Current smokers had lower odds of being overweight compared with non-smokers across all models, consistent with several previous studies (39–41). This well-documented association likely reflects the metabolic effects of nicotine on appetite suppression and energy expenditure, as well as potential reverse causality in this older adult cohort. Past smokers showed no significant association with overweight or obesity, although a borderline positive association with obesity was observed in Model 2, consistent with evidence that weight gain commonly follows smoking cessation (39–41). Given the low prevalence of past smoking in women (3%), sex-specific effects of smoking cessation on obesity could not be reliably examined in this study. Alcohol consumption frequency was not significantly associated with any of the three obesity outcomes after full adjustment, and its inclusion did not materially alter the observed socioeconomic gradient, suggesting that differential alcohol consumption does not explain the reversed household wealth pattern.

Educational attainment showed a complex and outcome-specific pattern. No significant association was found between education and overweight in any model. For obesity, participants with primary or less education had significantly higher odds in the fully adjusted model compared with university-educated participants (OR 1.56, 95% CI 1.03–2.34), consistent with the conventional inverse socioeconomic gradient observed in high-income settings where lower education is associated with higher obesity risk (12, 42, 43). For central obesity, education showed a significant inverse association in the minimally adjusted model that was fully attenuated after adjustment, suggesting confounding by residence and cardiometabolic factors. These findings are broadly consistent with studies reporting significant educational gradients in obesity, which are often more pronounced in women than in men (13, 43). One conventional explanation of the educational gradient in obesity is that unhealthy diets and lower levels of physical activity are more common among individuals with lower socioeconomic status (12, 44).

The ownership of household assets in this study was strongly and positively associated with the odds of overweight and obesity in a dose–response fashion, an association in the unexpected direction relative to findings from high-income countries. In the fully adjusted model, participants in the moderately deprived quartile (Q3) had significantly higher odds of both overweight (OR 1.68, 95% CI 1.00–2.80) and obesity (OR 2.05, 95% CI 1.35–3.11) compared with the most deprived quartile, and those in the least deprived quartile (Q4) had significantly higher odds of obesity (OR 1.88, 95% CI 1.09–3.25). A formal linear trend test confirmed a significant dose–response relationship for overweight (OR per quartile increase: 1.25, p for trend = 0.038) and obesity (OR per quartile increase: 1.30, p for trend = 0.002). By contrast, in most developed countries, obesity is widely considered a condition disproportionately affecting lower socioeconomic groups (12, 42). The reversed gradient observed here is consistent with findings from systematic reviews of low- and middle-income countries at early stages of nutrition transition, where access to calorie-dense foods and sedentary lifestyles first becomes concentrated among more affluent groups, before gradually reversing as economic development progresses and healthy behaviors become differentially adopted by higher-income groups (13, 42). For selected household items such as televisions, computers, and cars, the effect on obesity may be mediated by reduced physical activity, increased sedentary time, and higher dietary energy intake (45).

The divergence between household assets and education as socioeconomic indicators is scientifically meaningful in itself (46). Household asset ownership reflects post-independence economic stratification and current material living standards, while educational attainment in this cohort, born between 1938 and 1965, was largely completed during the Soviet era, a period characterized by relative socioeconomic homogeneity and centralized resource allocation (47). That household assets show a reversed gradient, while education shows a more conventional pattern, is consistent with evidence that different SES indicators capture distinct dimensions of socioeconomic position, with potentially different mechanisms of influence on health outcomes (46). In transitional-economy settings, material wealth may more directly reflect post-independence consumption patterns and lifestyle changes, while education, completed under a more equalized system, may operate through distinct biological and behavioral pathways. This highlights the importance of using multiple socioeconomic indicators in epidemiological studies in post-Soviet and transitional economy settings. The higher prevalence of overweight and obesity in non-ethnic Kazakhs may also be related to differences in lifestyle and nutrition across ethnic groups, but we do not have sufficient data to explore this important consideration further in this study.

Several mechanisms may explain the positive association between socioeconomic status and obesity observed in this study. Among socioeconomically disadvantaged groups, low or moderate food intake and high energy expenditure from manual labor are more common, whereas access to excessive amounts of calorie-dense food and adoption of sedentary lifestyles are more characteristic of more affluent groups in transitional economies (13, 42). In addition, certain cultural values in Central Asia may associate a larger physique with wealth and prosperity, as is commonly observed at early stages of socioeconomic development (18). The higher prevalence of overweight and obesity among non-ethnic Kazakhs in our study may also be related to differences in lifestyle, dietary patterns, and cultural norms across ethnic groups, though we do not have sufficient data to explore this important consideration further. Overall, consumption of healthy foods such as fruit and vegetables in the former Soviet Union appears inadequate, particularly among lower socioeconomic groups, which may partly explain why the expected inverse socioeconomic gradient, where lower socioeconomic status is associated with poorer dietary quality and higher obesity risk, has not yet fully emerged in this population (48).

The study has several limitations. First, the cross-sectional study design limits causal inference, and reverse causality cannot be excluded for some associations; in particular, household asset status may be influenced by health outcomes over time. Second, the study sample was drawn from one urban and one rural site in the Astana region and cannot be considered nationally representative of Kazakhstan, a geographically vast and ethnically diverse country; generalizations of prevalence estimates to the national population should be made with caution. Third, several behavioral determinants of obesity were not included in this analysis. Data on physical activity, occupational history, and dietary intake were not available for this analysis and represent limitations that future studies in this population should address. Alcohol consumption frequency data were available and included in all regression models; however, more detailed items on quantity and binge-drinking episodes had substantial missingness and were excluded. Fourth, the study’s response rate was modest (60%), similar to that of many contemporary population-based studies (49). Because respondents in epidemiological studies tend to have higher socioeconomic status and better health than non-respondents (50), and because recruitment through polyclinics may have attracted a less healthy and more health-motivated population, selection bias cannot be fully excluded. However, sensitivity analyses confirmed that participants included in the fully adjusted models did not differ significantly from those excluded due to missing data on key characteristics, suggesting that missing data-related bias was unlikely to materially affect the regression estimates. Fifth, while the data were collected between 2012 and 2015, this period was deliberately chosen to capture the last generation of Kazakhstani adults whose formative years were shaped by Soviet-era nutritional and socioeconomic conditions. This cohort cannot be reproduced, and the temporal specificity of this dataset is therefore a scientific strength uniquely suited to the research question rather than a limitation. At the same time, we acknowledge that obesity prevalence, urbanization patterns, and socioeconomic conditions in Kazakhstan may have evolved since data collection, and findings should be interpreted with this temporal context in mind when drawing inferences about present-day public health conditions.

Kazakhstan’s national non-communicable disease strategy and primary care polyclinic infrastructure provide a foundation for integrated obesity screening and management. The urban–rural disparity in central obesity prevalence (81.5% urban vs. 61.8% rural) suggests that urbanization-linked behavioral change is a key driver of the central adiposity burden, underscoring the need for urban planning policies that promote physical activity, including pedestrian infrastructure and green spaces, in rapidly growing cities such as Astana. The reversed socioeconomic gradient further suggests that obesity prevention strategies in Kazakhstan should not exclusively target socioeconomically disadvantaged groups, as is common practice in high-income country contexts, but should also address the behavioral and dietary patterns of more affluent urban populations. The strong and consistent associations between all obesity outcomes and diabetes, hypertension, and hypercholesterolemia underscore the urgent need for integrated obesity and cardiometabolic disease prevention within Kazakhstan’s primary care system.

In conclusion, the prevalence of obesity among older adults in this study is high, with over 80% of participants being overweight or obese and 72% having central obesity, accompanied by a distinct and non-conventional socioeconomic pattern. Household asset ownership showed a reversed socioeconomic gradient, with more affluent participants having significantly higher odds of obesity, consistent with Kazakhstan’s position as a transitional economy in the early stages of the nutrition transition. Educational attainment showed a more complex pattern, with lower education associated with higher odds of obesity, highlighting the importance of examining multiple dimensions of socioeconomic position in this context. The strong and consistent associations between all three obesity outcomes and diabetes, hypertension, and hypercholesterolemia underscore the urgent need for integrated obesity and cardiometabolic disease prevention strategies in Kazakhstan. Rural residence was the strongest protective factor against central obesity, suggesting that urbanization-linked behavioral changes are a key driver of the central adiposity burden. These findings call for context-specific public health approaches that account for the distinct socioeconomic patterning of obesity in Kazakhstan, target both urban and rural populations, and prioritize integrating obesity prevention into Kazakhstan’s broader non-communicable disease control strategy.

## Data Availability

The raw data supporting the conclusions of this article will be made available by the authors, without undue reservation.

## Supplementary material

SUPPLEMENTARY FIGURE 1 Predicted probability of overweight, obesity, and central obesity by household asset quartile among older adults in the Astana region of Kazakhstan. Predicted probabilities were estimated from fully adjusted binary logistic regression models with household asset quartile treated as a continuous variable. Models were adjusted for age, sex, diabetes, hypertension, hypercholesterolemia, smoking status, alcohol consumption frequency, place of residence, marital status, education, and ethnicity. Shaded areas represent 95% confidence intervals. Q1, most deprived; Q2, partially deprived; Q3, moderately deprived; Q4, least deprived.
