# Supplementary material for: Socioeconomic inequalities in obesity among older adults in the Astana region of Kazakhstan: evidence of a reversed socioeconomic gradient
Source: Front Public Health. 2026 Jul 10;14:1895120. doi: 10.3389/fpubh.2026.1895120 (PMC13395783; doi:10.3389/fpubh.2026.1895120)
Supplement: Supplementary file 1 [file Data_Sheet_1.pdf]

Supplementary Table 1. Comparison of participants included and excluded from fully adjusted regression models

Characteristic	Overweight / Obesity Models	Overweight / Obesity Models	P-value	Central Obesity Model	Central Obesity Model	P-value
	Included (n = 854)	Excluded (n = 123)		Included (n = 852)	Excluded (n = 125)	
**Education, n (%)**			0.492			0.468
Higher education	255 (29.86)	33 (27.73)		255 (29.93)	33 (27.27)	
Vocational/apprenticeship	295 (34.54)	37 (31.09)		294 (34.51)	38 (31.40)	
Primary/secondary	304 (35.60)	49 (41.18)		303 (35.56)	50 (41.32)	
**Household asset quartile, n (%)**			0.361			0.327
Q1 Most deprived	211 (24.71)	19 (25.33)		210 (24.65)	20 (25.97)	
Q2 Partially deprived	245 (28.69)	24 (32.00)		244 (28.64)	25 (32.47)	
Q3 Moderately deprived	265 (31.03)	26 (34.67)		265 (31.10)	26 (33.77)	
Q4 Least deprived	133 (15.57)	6 (8.00)		133 (15.61)	6 (7.79)	
**Ethnicity, n (%)**			0.373			0.225
Kazakh	501 (58.67)	65 (54.17)		502 (58.92)	64 (52.46)	
Russian	219 (25.64)	38 (31.67)		217 (25.47)	40 (32.79)	
Other	134 (15.69)	17 (14.17)		133 (15.61)	18 (14.75)	
**Place of residence, n (%)**			0.690			0.837
Urban	433 (50.70)	60 (48.78)		431 (50.59)	62 (49.60)	
Rural	421 (49.30)	63 (51.22)		421 (49.41)	63 (50.40)	
**BMI/WHR category, n (%)***			0.936			0.544
Normal weight / Normal WHR	162 (18.97)	23 (20.00)		244 (28.64)	29 (25.89)	
Overweight / High WHR	318 (37.24)	41 (35.65)		608 (71.36)	83 (74.11)	
Obesity	374 (43.79)	51 (44.35)				
**Sex, n (%)**			0.612			0.472
Male	375 (43.91)	57 (46.34)		373 (43.78)	59 (47.20)	
Female	479 (56.09)	66 (53.66)		479 (56.22)	66 (52.80)	
**Age group, n (%)**			0.712			0.594
50-54 years	233 (27.28)	31 (25.20)		233 (27.35)	31 (24.80)	
55-59 years	200 (23.42)	24 (19.51)		200 (23.47)	24 (19.20)	
60-64 years	174 (20.37)	27 (21.95)		174 (20.42)	27 (21.60)	
65-69 years	127 (14.87)	19 (15.45)		126 (14.79)	20 (16.00)	
70-74 years	120 (14.05)	22 (17.89)		119 (13.97)	23 (18.40)	

BMI: body mass index; WHR: waist-to-hip ratio.
** BMI category n totals 969; WHR category n totals 964, due to missing anthropometric values.*
p-values from Pearson chi-square tests.
Excluded participants had missing data on one or more covariates included in the fully adjusted model.
$p<0.05$ was considered statistically significant.
Excluded participants had missing data on the outcome itself or on one or more covariates in the fully adjusted model.

Supplementary Table 2. Model fit statistics for fully adjusted logistic regression models

Outcome	n	Pseudo R^2	Hosmer-Lemeshow χ^2 (df = 8)	HL p-value	AUC
Overweight (BMI ≥25 kg/m²)	854	0.136	8.81	0.359	0.747
Obesity (BMI ≥30 kg/m²)	854	0.102	4.51	0.808	0.712
Central obesity (WHR >0.90 men / >0.85 women)	852	0.130	6.13	0.633	0.740

HL: Hosmer-Lemeshow; AUC: area under the receiver operating characteristic curve; BMI: body mass index; WHR: waist-to-hip ratio.
Pseudo R^2 refers to McFadden's pseudo R^2.
All models were fully adjusted for age, sex, diabetes, hypertension, hypercholesterolemia, smoking status, alcohol consumption frequency, residence, marital status, education, ethnicity, and household asset quartile.
A p-value of 0.05 in the Hosmer-Lemeshow test indicates adequate model fit.
AUC values between 0.70 and 0.80 indicate acceptable discriminative ability.
$p<0.05$ was considered statistically significant.

Supplementary Table 3. Linear trend test for association between household asset quartile and obesity outcomes

Outcome	OR per Quartile Increase	95% CI	p for Trend
Overweight (BMI ≥25 kg/m²)	1.25	1.01-1.55	0.038
Obesity (BMI ≥30 kg/m²)	1.30	1.10-1.54	0.002
Central obesity (WHR >0.90 men / >0.85 women)	1.04	0.86-1.25	0.685

OR: odds ratio; CI: confidence interval; BMI: body mass index; WHR: waist-to-hip ratio.
Each model was fully adjusted for age, sex, diabetes, hypertension, hypercholesterolemia, smoking status, alcohol consumption frequency, residence, marital status, education, and ethnicity, with household asset quartile treated as a continuous variable.
Robust standard errors were used throughout.
$p<0.05$ was considered statistically significant.

Supplementary Table 4. Association between household asset quartile and obesity outcomes in models excluding cardiometabolic covariates (diabetes, hypertension, and hypercholesterolemia)

Household Asset Quartile	Overweight OR (95% CI)	Obesity OR (95% CI)	Central Obesity OR (95% CI)
Q1 (most deprived)	1.00 (Ref)	1.00 (Ref)	1.00 (Ref)
Q2 Partially deprived	1.54 (0.96-2.46)	1.53 (1.04-2.27)*	0.98 (0.65-1.49)
Q3 Moderately deprived	1.91 (1.16-3.15)*	1.90 (1.28-2.83)**	1.08 (0.70-1.68)
Q4 (least deprived)	2.28 (1.15-4.49)*	1.96 (1.16-3.30)*	1.39 (0.74-2.62)

OR: odds ratio; CI: confidence interval.
Models adjusted for age, sex, smoking status, alcohol consumption frequency, place of residence, marital status, educational attainment, ethnicity, and household asset quartile. Diabetes, hypertension, and hypercholesterolemia were excluded to assess potential overadjustment.
Robust standard errors used throughout.
$^*p<0.05$; $^{**}p<0.01$
