# Supplementary figures and images for: Socioeconomic inequalities in obesity among older adults in the Astana region of Kazakhstan: evidence of a reversed socioeconomic gradient
Source: Front Public Health. 2026 Jul 10;14:1895120. doi: 10.3389/fpubh.2026.1895120 (PMC13395783; doi:10.3389/fpubh.2026.1895120)

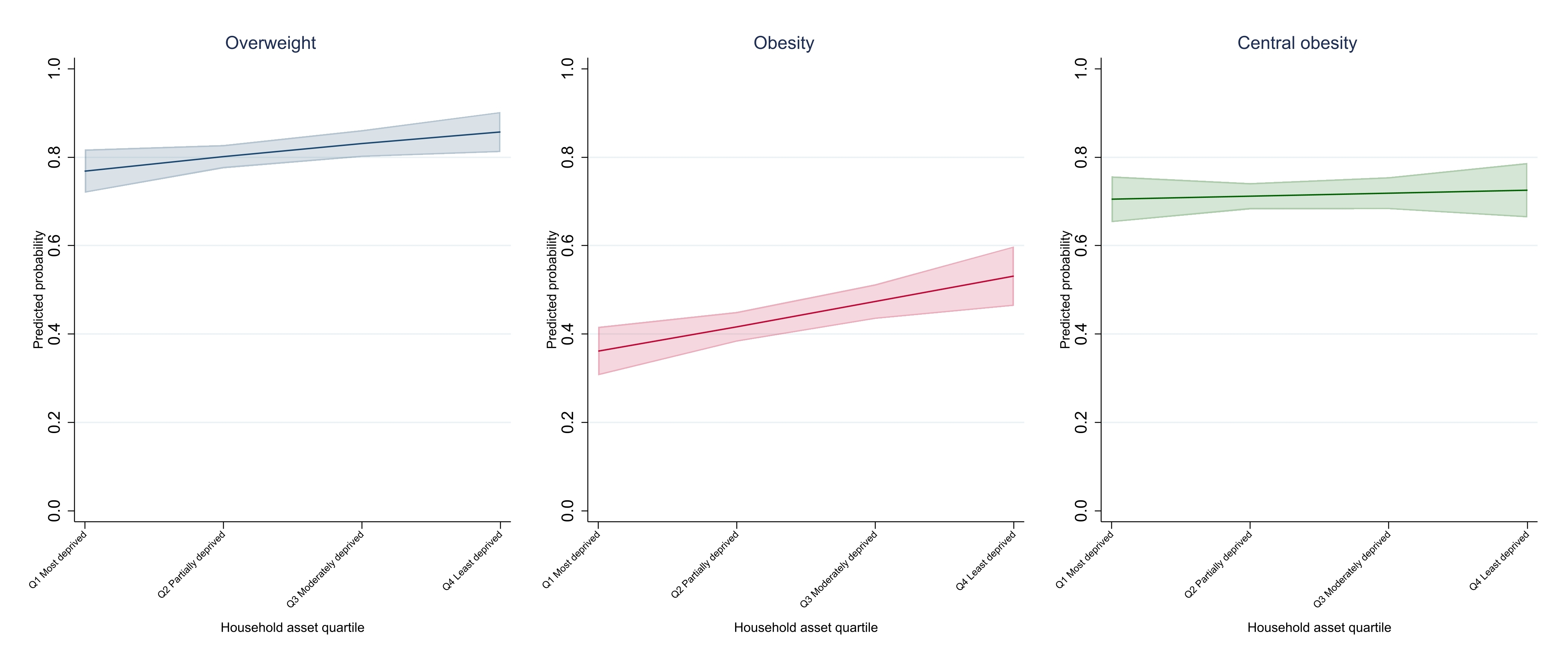

Supplement: SUPPLEMENTARY FIGURE 1 — Predicted probability of overweight, obesity, and central obesity by household asset quartile among older adults in the Astana region of Kazakhstan. Predicted probabilities were estimated from fully adjusted binary logistic regression models with household asset quartile treated as a continuous variable. Models were adjusted for age, sex, diabetes, hypertension, hypercholesterolemia, smoking status, alcohol consumption frequency, place of residence, marital status, education, and ethnicity. Shaded areas represent 95% confidence intervals. Q1, most deprived; Q2, partially deprived; Q3, moderately deprived; Q4, least deprived. [file Image_1.jpeg]
